# Right hydrocelectomy with right orchidectomy in a case of right hydrocele since last 15 years

**DOI:** 10.11604/pamj.2022.42.291.35635

**Published:** 2022-08-18

**Authors:** Sheetal Asutkar, Suraj Rathod

**Affiliations:** 1 Department of Shalyatantra, Mahatma Gandhi Ayurved College Hospital and Research Centre, Salod (Hirapur), Wardha, Maharashtra, India

**Keywords:** Orchidectomy, hydrocelectomy, necrosis, hemostasis

## Image in medicine

A 72-year-old male patient came to the outpatient department (OPD) of Mahatma Gandhi Ayurveda College, Hospital and Research Center, Salod, Wardha with a complaint of swelling in the scrotum and buried penis for 15 years. On examination, it was found to be a right-sided huge hydrocele with a hard sac of a coconut size with trans-illumination test negative and folding test positive. On ultrasonography, it was reported to be a huge hydrocele (bilateral). All hemodynamic parameters were within normal limits. The patient has advised surgery (right hydrocelectomy with orchidectomy) under spinal anesthesia. After taking orchidectomy consent patient was posted for surgery. The patient was operated upon with an incision parallel to the median raphe and after adequate dissection of the huge sac within the layers of the scrotum, the whole sac of the right hydrocele was excised with drainage of dirty white fluid from the most fluctuant part. Gross calcification of the hydrocele sac was observed with gross necrosis of the right testis. Right hydrocelectomy with orchidectomy was performed, and the scrotum was closed in layers with an assurance of complete hemostasis. After the procedure, the hydrocelectomy sac was dissected for visualizing the contents and the whole calcified hard sac with necrotic testes and dirty white fluid was found. The specimen was sent for histopathological evaluation. The patient was kept in the ward for seven days for post-operative management. He had a low-grade fever on the first post-operative day. Given intravenous antibiotics for five days and other medications like analgesics Intravenous fluids and scrotal support. The patient was discharged after seven days and later called for stitch removal after five days. Patient is absolutely free of symptoms now.

**Figure 1:**
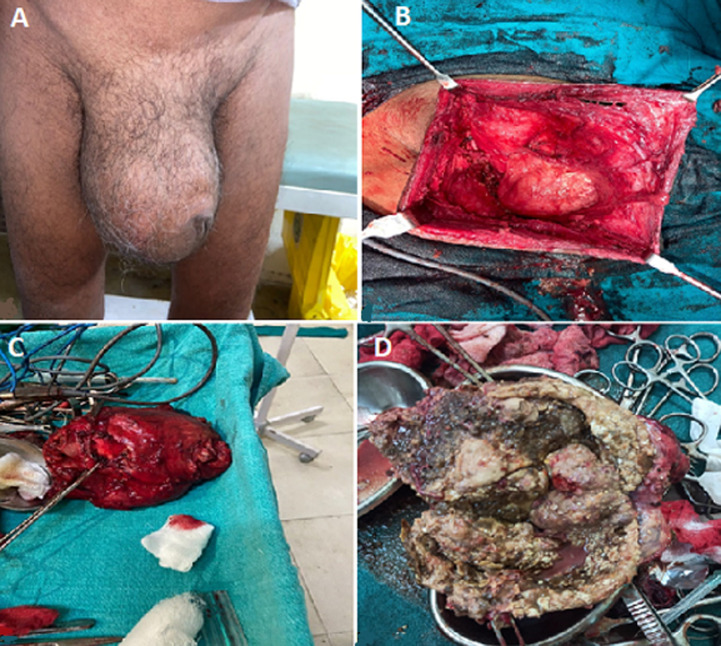
A) right chylocele; B) right carcinoma of testis; C) right hematocele; D) right inguinal hernia

